# Metabolic analysis of MYB30 that regulates iron deficiency stress in *Arabidopsis*

**DOI:** 10.3389/fpls.2026.1756499

**Published:** 2026-02-23

**Authors:** Qianyuan Gong

**Affiliations:** Medical Research Center, The Affiliated Hospital of Southwest Jiaotong University, The Third People’s Hospital of Chengdu, Chengdu, Sichuan, China

**Keywords:** iron deficiency stress, metabolic pathway, metabolomic analysis, microelement, transcription factor

## Abstract

**Introduction:**

Iron is an essential microelement for animals, humans, and plants. Notably, approximately one-third of the world’s soils are alkaline, leading to iron deficiency. Therefore, understanding the mechanism of iron absorption and transport in plants is crucial for improving iron bioavailability in crops.

**Methods:**

In this research, reverse genetics was used to identify the transcription factor MYB30 as a positive regulator of the plant response to iron deficiency.

**Results and Discussion:**

Phenotype analysis demonstrated that MYB30 mutant plants were sensitive to iron deficiency, exhibiting reduced root length, lower chlorophyll content, and elevated lipid peroxidation, whereas MYB30 overexpression lines showed enhanced tolerance. Metabolomic analysis of myb30 plant roots by mass spectrometry indicated decreased antioxidant activity and detoxification capacity under iron-deficient conditions. Interestingly, 22 metabolic pathways were altered in the *myb30* plant under iron deficiency. This metabolic reprogramming likely compromises plant growth. Furthermore, MYB30 reduced reactive oxygen species accumulation under iron deficiency stress by activating related genes and enhancing antioxidant enzyme activity. In summary, metabolite analysis provides detailed molecular insights into plant iron deficiency stress and supports molecular genetic breeding efforts to improve mineral nutrition in crops.

## Introduction

Plants absorb essential nutrient elements from the soil through their root hairs to support growth and reproduction (Wang et al., 2018). Iron (Fe) is a vital microelement involved in many physiological processes, including but not limited to photosynthesis, protein and nucleic acid synthesis, respiration, and electron transfer (Ladygin, 2004; Zhang et al., 2019). However, iron availability is limited in calcareous and alkaline soils, which constitute approximately one-third of the world’s cultivated land, posing a challenge for plant growth (Korcak, 2011).

In response to iron deficiency, plants have developed two major regulatory mechanisms: strategy I, employed by dicotyledonous and non-gramineous plants, and strategy II, specific to gramineous species (Ivanov et al., 2012). Strategy I involves physiological responses in plant roots, such as rhizosphere acidification, increased ferric reductase activity, and enhanced Fe^2+^ transport through the activity of H^+^-ATPase, ferric reduction oxidase, and iron-regulated transporter (IRT) expression (Brumbarova et al., 2015; Schikora and Schmidt, 2002; Santi et al., 2005). These responses are regulated by transcription factors such as *FIT*, *bHLH38/39*, *bHLH100/101*, and *PYE* (Colangelo and Guerinot, 2004; Wang et al., 2013). In strategy II, plants secrete iron chelators, such as phytosiderophores, to facilitate iron uptake (Jeong and Guerinot, 2009).

Recent investigations have shown that other transcription factors, including WRKYs, NACs, and MYBs, also regulate the response to iron deficiency (Kobayashi et al., 2007; Yan et al., 2016). WRKY46 rapidly responds to iron deficiency by upregulating the iron transporter gene *VITL1*, thereby promoting iron translocation from roots to shoots (Huang et al., 2024). The MYB domain in MYBs is highly conserved and typically consists of one to four amino acid repeats (R), classifying the MYB family into R1R2, R2R3, and R1R2R3 types (Liu et al, 2015). Some MYB family members, such as MYB10 and MYB72, activate nicotinamide synthase gene expression under iron-deficient conditions (Palmer et al., 2013). In contrast, MxMYB1 has a negative regulatory effect on iron uptake and preservation in plants (Shen et al., 2008).

In the present study, we report that MYB30, a member of the R2R3-MYB family, positively affects the plant’s response to iron deficiency. Phenotypic analysis demonstrated that the *myb30* plants were sensitive to iron-deficient conditions, showing lower chlorophyll content, shorter root length, and elevated lipid peroxidation, while MYB30 overexpression plants displayed a reverse trend. Moreover, we conducted an extensive metabolomics analysis of *myb30* plants under iron-deficient conditions using gas chromatography–mass spectrometry (GC-MS).

## Materials and methods

### Mutants and overexpression plants

Seeds of *Arabidopsis thaliana* Columbia-0 (WT), *myb30-1* (SALK_061315), and *myb30-2* (SALK_027644C) (a T-DNA insertion in the 5′-UTR of *At3g28910* in the Col-0 background) were sourced from the Biological Resource Center of *Arabidopsis*. *Pro35S:MYB30-Flag*/Col-0 transgenic lines were constructed by transforming the *Pro35S:MYB30-Flag* vector into Col-0 via *Agrobacterium* strain EHA105-mediated infiltration. Two independent transgenic lines in T2 progeny, MYB30-OX 1 and MYB30-OX 2, were used in the present research. The related primers are shown in **Supplementary Table 1**.

### Plant phenotypic analysis

The seeds of the plants were sterilized on the surface using 70% ethanol, rinsed with sterile distilled water, and then planted on Murashige and Skoog (MS) culture with 2.5% sucrose and 0.3% phytagel agar (Sigma-Aldrich, St. Louis, USA). Plates were kept at 4 °C for 2 days and then transferred to constant illumination at 23°C. For phenotypic assays, 4-day-old plants were vertically grown on MS culture (pH = 5.8) with or without iron (36.7 mg/L of FeNaEDTA). MS culture (Cat PM1011) and MS-Fe (Cat PM1061-Fe) culture were purchased from Coolaber Science & Technology (Beijing, China). Photographs were taken and root length was scored after 5 days of growth using ImageJ (https://imagej.nih.gov/ij/).

### Chlorophyll content and Fe concentration determination

Chlorophyll content was measured using previously described methods (Arnon, 1949). Briefly, plant shoots were ground to a powder in liquid nitrogen and then resuspended in 80% (v/v) acetone and centrifuged at 10,000*g* at 4 °C for 10 min. The spectroscopy absorbance was measured at 646 and 663 nm.

For Fe concentration determination, plant roots and leaves were dried at 70 °C for 3 days. Approximately 0.1 g of dried tissue was digested with a SpeedWave Two (Berghof Products, Eningen, Germany) using a mixture of hydrogen peroxide and HNO_3_ (4:1, v:v) at 200 °C for 60 min. Fe concentration was measured by atomic emission spectroscopy.

### Measurement of the content of malondialdehyde

Malondialdehyde (MDA) was determined using a plant MDA assay kit (Solarbio, Beijing, China, Cat BC0025). Following the kit’s instructions, approximately 0.5 g of the sample was weighed, and 0.5 mL of the extraction solution was added for homogenization in an ice bath. The mixture was then centrifuged at 8,000*g* for 10 min at 4°C. One hundred microliters of the supernatant was taken, and 300 μL of MDA detection working solution and 100 μL of reagent 3 were added, mixed well, incubated in a 100°C water bath for 60 min, cooled to room temperature, and centrifuged at 10,000*g* for 10 min. The absorbance of each sample was measured at 450, 532, and 600 nm, and the content of MDA was calculated. In the control tube, 100 μL of distilled water was added for the reaction.

### H_2_O_2_ histochemical detection and content determination

Reactive oxygen species (ROS) were measured in terms of H_2_O_2_ levels. For H_2_O_2_ histochemical detection, seedlings were incubated overnight in the dark with 1 mg/mL of 3,3′-diaminobenzidine (DAB) solution (pH = 3.8). Seedlings were immersed in 95% ethanol for 12 h to remove chlorophyll.

H_2_O_2_ levels were assessed using a Solarbio (Beijing, China) H_2_O_2_ Content Assay Kit (Cat<ns/> BC3595). Approximately 50 mg of fresh tissue sample was powdered with 500 μL of cold acetone. Following a 10-min centrifugation at 8,000*g* at 4 °C, the supernatant was extracted for examination. A 250-μL aliquot of the supernatant was taken from the test tube, and 25 μL of reagent 2 and 50 μL of reagent 3 were added, after which the mixture was reacted in a room-temperature water bath for 10 min. The sample was then centrifuged at 4,000*g* for 10 min, and the supernatant was discarded. Subsequently, 250 μL of reagent 4 was added to dissolve the precipitate, and the absorbance value was measured at a wavelength of 415 nm to calculate the H_2_O_2_ content. For the control tube, 250 μL of reagent 1 was added.

### Antioxidant enzymatic activity

Enzymatic antioxidant activity was determined in 4-day-old seedlings with or without iron deficiency treatment for further growth for an additional 5 days. The activities of peroxidase (POD, Cat A084-3-1), superoxide dismutase (SOD, Cat A001-4), and catalase (CAT, Cat A007-1-1) were determined using assay kits from Jiancheng (Nanjing, China) following the kit’s instructions.

For POD detection, approximately 0.1 g of fresh tissue was taken, and 9 times the volume of physiological saline was added according to a weight (g) to volume (mL) ratio of 1:9. Then, 10% tissue homogenate was prepared under an ice-water bath and centrifuged at 3,000 rpm for 10 min. Next, 0.1 mL of the supernatant was taken, and 2.4 mL of reagent 1, 0.3 mL of reagent 2, and 0.2 mL of reagent 3 were added. After reaction at 37 °C for 30 min, 1 mL of reagent 4 was added to terminate the reaction. The supernatant was then taken, and the absorbance was measured at 420 nm to calculate the enzyme activity. In the control tube, reagent 3 was omitted. For CAT detection, after the tissue was homogenized, 0.1 mL of the supernatant was taken, and 1 mL of pre-warmed reagent 1 at 37 °C and 0.1 mL of reagent 2 were added. After reacting for 1 min, 1 mL of reagent 3 and 0.1 mL of reagent 4 were added and mixed well, and the absorbance at 405 nm was measured to calculate the enzyme activity. The control tube was finally added with the tissue homogenate. For SOD detection, 0.2 g of fresh tissue was taken, and 4 times the volume of homogenization medium (reagent 8) was added. A 20% tissue homogenate was prepared under an ice-water bath and centrifuged at 3,500 rpm for 10 min. A 0.1-mL aliquot of the supernatant was taken and diluted 3 times with reagent 8. Then, 60 μL of the diluted solution was taken, and 1 mL of reagent 1, 0.1 mL of reagent 2, 0.1 mL of reagent 3, and 0.1 mL of reagent 4 were added, mixed well, and reacted at 37 °C for 40 min. Two milliliters of chromogenic reagent was added, and the absorbance was measured at 550 nm to calculate the enzyme activity. The control tube was added with the corresponding volume of reagent 8 for the reaction.

### Metabolite profiling and data analysis

Plant metabolites were extracted from the roots of 4-day-old WT and *myb30–2* seedlings with or without iron deficiency stress for an additional 5 days for further growth, quantified by GC-MS time-of-flight (TOF) and analyzed as described previously (Watanabe et al., 2013), using the RBioconductor package gpfortify to construct heat maps and principal component analysis (PCA). Metabolites that showed significant changes were defined as previously described (Benjamini and Hochberg, 1995). Metabolomics pathway analysis (MPA) was calculated from relative concentrations after removing non-changing data between WT and *myb30–2* lines under iron-deficient conditions.

### Reverse transcription-quantitative PCR assays

Total RNA was isolated from treated seedlings using TRIzol reagent from TianGen (Beijing, China). One microgram of this RNA was employed for reverse transcription with M-MLV reverse transcriptase (Vazyme, Nanjing, China). The gene expression levels were determined using the SYBR quantitative PCR Mix on a Roche quantitative instrument (Rotkreuz, Switzerland). Relative mRNA levels were assessed using the 2^–ΔΔCt^ method, with *ACTIN2* serving as the internal reference. The primers are listed in **Supplementary Table 1**.

### Western blotting

Seedling proteins were extracted in RIPA lysate buffer (Biosharp, Hefei, China), separated via SDS-PAGE, and transferred to a PVDF membrane at 80 V for 2 h. Blots were treated with Flag antibody (1:4,000, Cell Signaling Technology, Danvers, USA) overnight at 8°C and then incubated with the secondary antibody (horseradish peroxidase-conjugated) for 1 h at 25°C, using a chemiluminescence reagent to visualize the proteins.

### Statistical analysis

One-way ANOVA test was used to analyze the statistical significance, with lowercase letters indicating significant differences (*P* < 0.05). The results are displayed as means ± SD. Root length measurements were assessed through nine seedlings per group (*n* = 9). For measuring chlorophyll content, Fe concentration, MDA content, and ROS content, approximately 0.5 g of shoots per group (*n* = 3) were used.

## Results

### MYB30 is involved in iron deficiency stress in Arabidopsis

**Figure 1:**
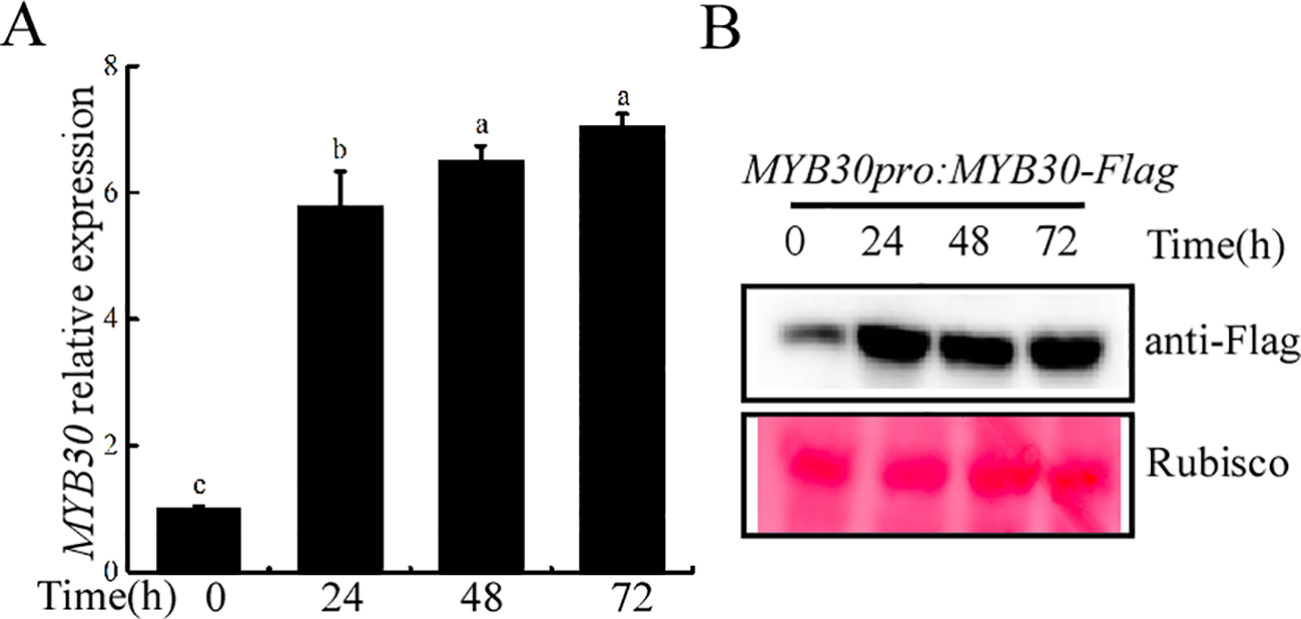
The expression of MYB30 was induced by iron-deficient treatment. **(A)** Expression analysis of the *MYB30* gene. RT-qPCR analysis of *MYB30* transcript levels in 4-day-old Col-0 seedlings that were used as control or were transferred to an iron-deficient medium for the indicated times. The data were adjusted based on gene expression levels in Col-0 under normal conditions. Lowercase letters above the bars indicated significant differences (*P* < 0.05) by one-way ANOVA. Error bar represents SD (*n* = 3). The experiment was repeated biologically three times. **(B)** Western blotting assays for MYB30 abundance. Four-day-old *MYB30pro:MYB30-Flag*/Col-0 seedlings that were transferred to control or iron-deficient medium for the indicated times. The total proteins were detected with anti-Flag and using Ponceau staining as loading controls.

To molecularly verify the involvement of MYB30 in the plant response to iron deficiency stress, 4-day-old seedlings of Col-0 were transferred to iron-deficient MS medium for further growth observation. Using fluorescence qRT-PCR, we found that the transcription level of the *MYB30* gene was elevated under iron-deficient conditions and increased progressively with treatment time. After 3 days of iron deficiency treatment, *MYB30* expression increased approximately sevenfold. Consistent with this, MYB30 protein abundance also increased significantly with prolonged iron deficiency treatment (**Figure 1**). These results indicate that MYB30 is activated at both the transcriptional and translation levels under iron deficiency, suggesting its potential role in the plant’s response to this stress.

### MYB30 positively regulates the response to iron deficiency stress

**Figure 2:**
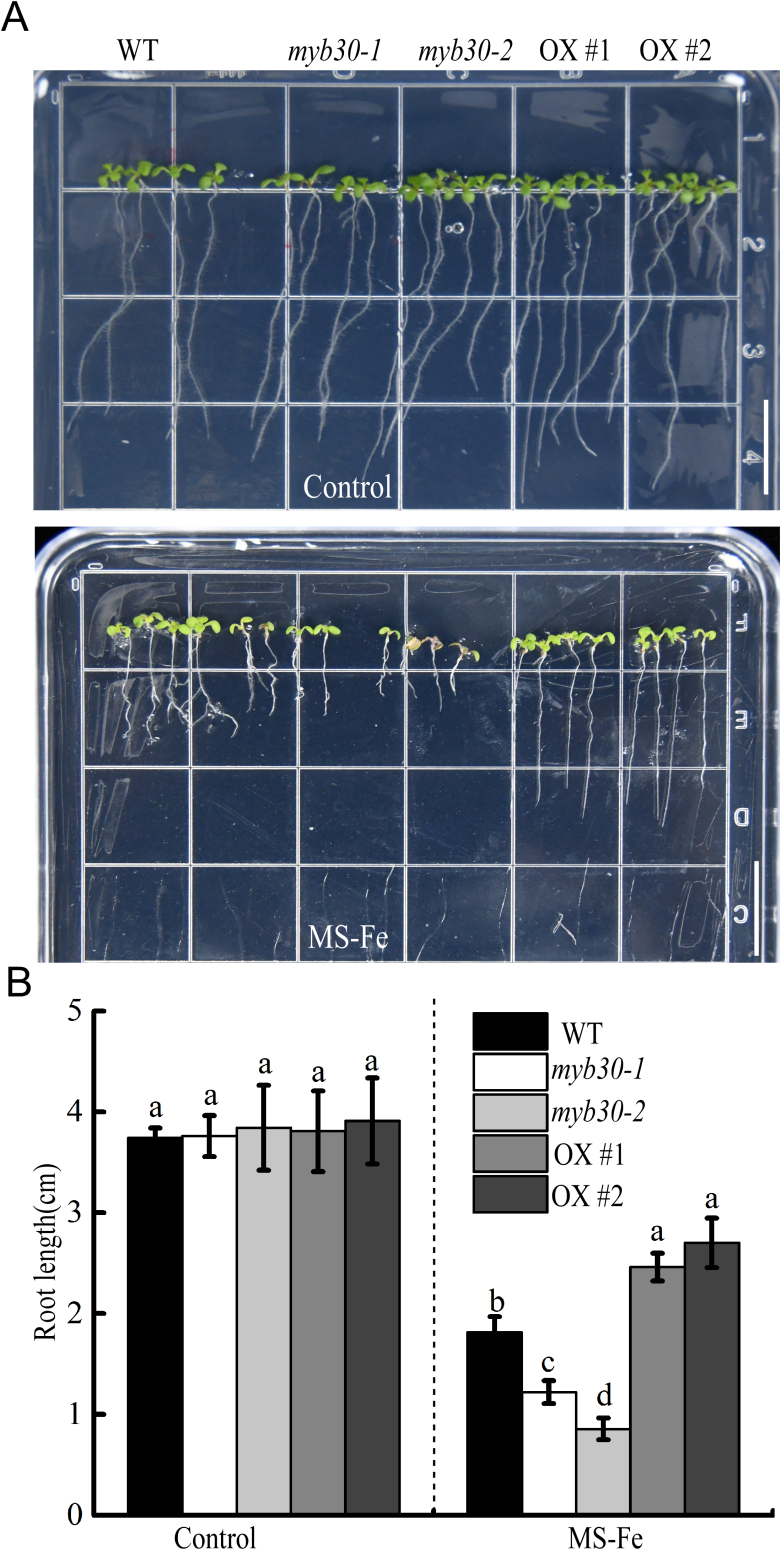
MYB30 is involved in plant iron deficiency stress response. Iron deficiency phenotypes analysis. Seedlings that were 4 days old were moved to either control or iron-deficient medium for further growth, and pictures were taken 5 days post-transfer. Bar = 1.5 cm. **(B)** The root length statistics of related materials are depicted in **(A)**. Lowercase letters above the bars indicated significant differences (*P* < 0.05) by one-way ANOVA. Error bar represents SD (*n* = 9). The experiment was repeated biologically three times.

To investigate the regulatory role of MYB30 under iron-deficient conditions, we examined the phenotypes of Col-0 and MYB30 loss-of-function mutants (*myb30–1* and *myb30-2*; **Supplementary Figure 1A**). When grown on standard MS medium, both mutants displayed growth similar to Col-0. Under iron-deficient conditions, however, all plants developed smaller leaves and shorter primary roots (**Figure 2**). Notably, the length of the primary roots of *myb30–1* and *myb30-2* was significantly shorter than that of Col-0, indicating enhanced sensitivity to iron deficiency.

To further confirm the role of *MYB30*, we used MYB30 overexpression lines (MYB30-OX, 1 and 2; **Supplementary Figure 1B**). Under sufficient iron, these lines grew similarly to the control. Under iron deficiency, however, their primary roots were significantly longer than those of Col-0 (**Figure 2**). Collectively, these results showed that MYB30 positively influences the plant response to iron deficiency stress, primarily by promoting root growth to enhance adaptation to low-iron environments.

### MYB30 modulates chlorophyll content in iron deficiency stress

**Figure 3:**
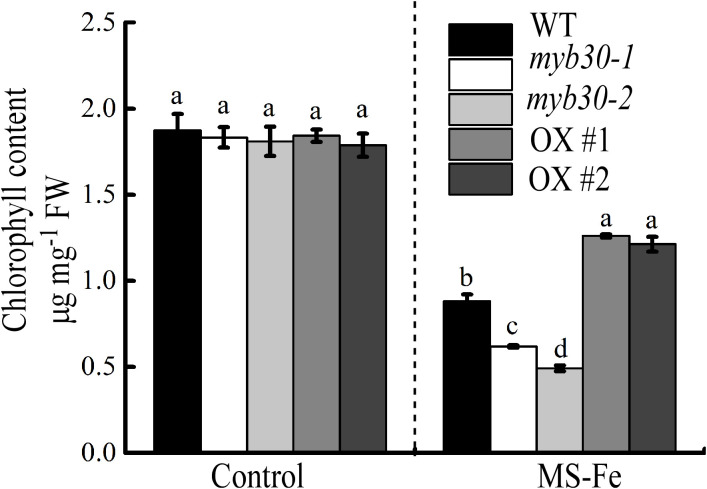
MYB30 modulates chlorophyll accumulation under iron-deficient treatment. Quantification of chlorophyll content in Col-0, *myb30*, and MYB30-OX seedlings. Seedlings that were 4 days old were moved to either control or iron-deficient medium for an additional 5 days for further growth, and the accumulation of chlorophyll in plants was determined. Lowercase letters above the bars indicated significant differences (*P* < 0.05) by one-way ANOVA. Error bar represents SD (*n* = 3). The experiment was repeated biologically three times. FW, fresh weight.

Iron deficiency leads to incomplete chloroplast structure, decreases photosynthetic capacity, and eventually causes huge losses in crop quality and yield (Ladygin, 2004). To assess whether MYB30 influences chlorophyll accumulation under iron deficiency, we measured chlorophyll content in plant shoots. All seedlings showed reduced chlorophyll content under iron deficiency, but the decrease was most pronounced in *myb30* plants. In contrast, MYB30-OX plants maintained higher chlorophyll levels (**Figure 3**). This suggests that MYB30 improves chlorophyll accumulation under iron deficiency.

### MYB30 is involved in lipid peroxidation in response to iron deficiency

**Figure 4:**
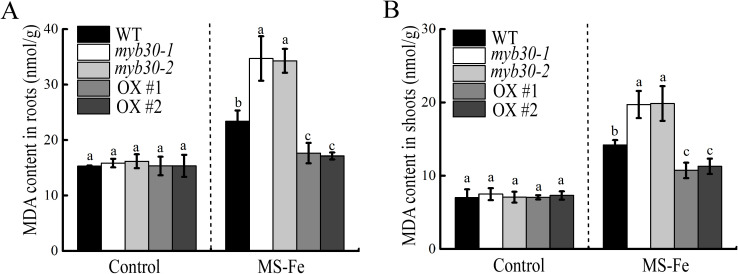
MYB30 is implicated in lipid peroxidation in response to iron deficiency. **(A)** MDA content in roots. **(B)** MDA content in shoots. Seedlings that were 4 days old were transferred to either control or iron-deficient media for an additional 5 days for further growth, and the MDA content was determined. Lowercase letters above the bars indicated significant differences (*P* < 0.05) by one-way ANOVA. Error bar represents SD (*n* = 3). The experiment was repeated biologically three times.

Although essential, iron can also be toxic due to its reactivity (Morrissey and Guerinot, 2009). ROS production is directly related to lipid peroxidation (Ma et al., 2015), which can be assessed by detecting MDA levels (Blokhina et al., 2003). Under iron deficiency, MDA content in the roots and shoots was significantly higher in *myb30* plants compared to the control, while MYB30-OX plants displayed a reverse trend (**Figure 4**). This indicates greater oxidative damage in *myb30* plants under iron-deficient conditions.

Together, these findings show that *myb30* plants exhibit reduced root elongation, lower chlorophyll content, and increased lipid peroxidation under iron deficiency. To uncover the mechanisms behind these responses, further metabolomics profiles were analyzed.

### The metabolite profile is changed by iron deficiency in plants

**Figure 5:**
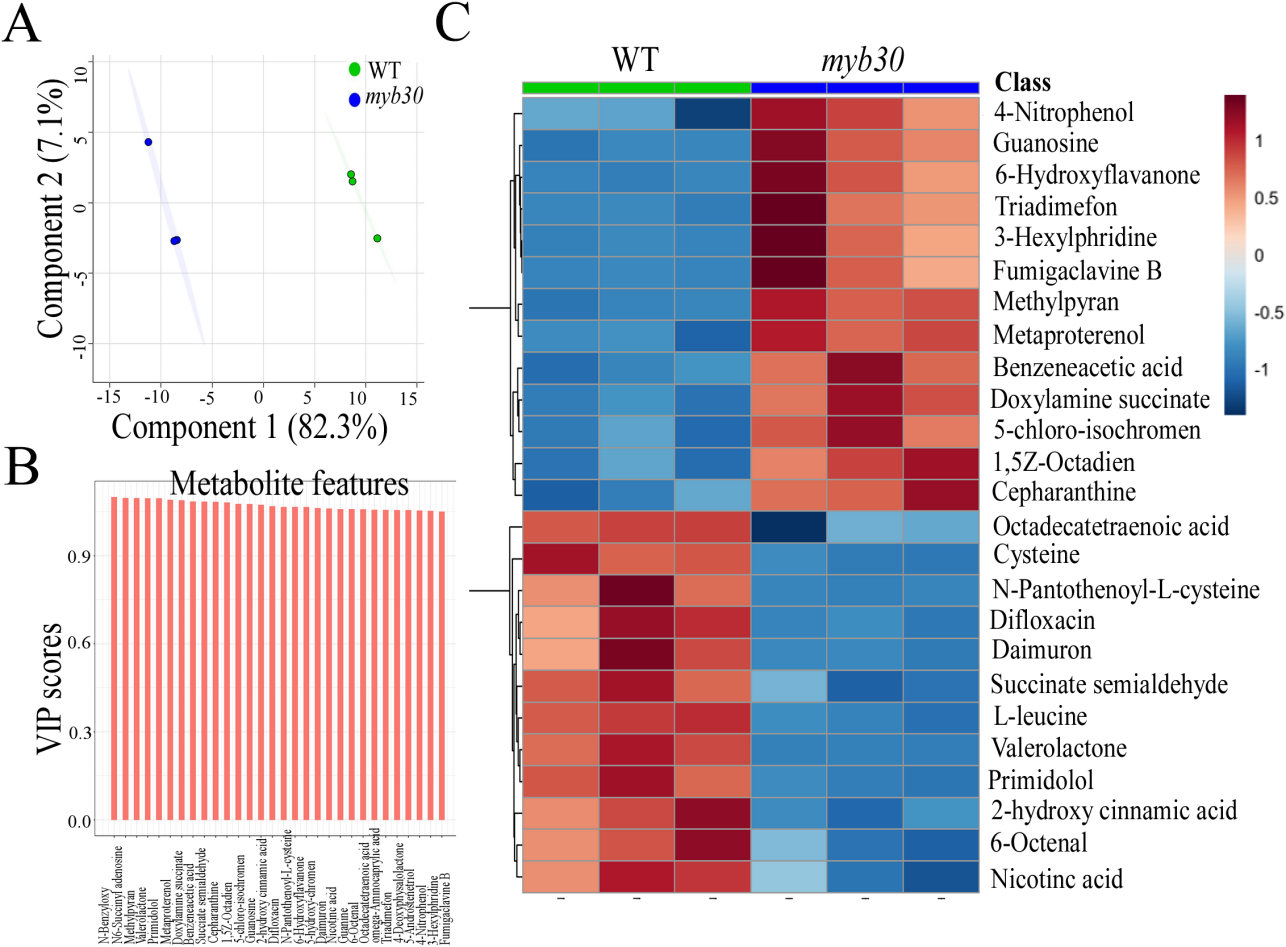
Metabolic profiling of WT and *myb30* plant roots grown under Fe deprivation. **(A)** Principal component analysis (PCA) of metabolite compositions of WT and *myb30* plant roots grown under Fe deficiency conditions. The percentage of total variance accounted for by each component is indicated. The *x*-axis represents the first principal component, while the *y*-axis represents the second. The dots represent experimentally related samples, and the color represents different groups. The more the samples within a group are clustered, the more dispersed the samples between groups are, and the more reliable the results will be. **(B)** Variable importance for the projection (VIP) scores from orthogonal projections to latent structures discriminant analysis (OPLS-DA) of plant roots showing the discriminating metabolites between WT and *myb30* seedlings under Fe deficiency conditions. VIP is used to explain the importance of the *X* dataset and the related *Y* dataset. The sum of the squares of all VIP values is equal to the total number of variables in the model, so its average value is 1. A variable is important when its VIP is ≥1—this is often used as a screening criterion for potential biomarkers. **(C)** A heatmap based on metabolite component hierarchical clustering. Numerical values represent the *z*-score of the log2-transformed levels (relative to the endogenous standard) for each identified metabolite. Hierarchical clustering was performed using the Ward D2 method (see *Experimental procedures*). The experiment was repeated biologically three times.

Using GC-MS metabolite profiling, we evaluated metabolomic changes between the Col-0 and *myb30* plants under iron deficiency. Among the 840 metabolites present in all samples, 134 were identified (**Supplementary Table 2**). To analyze the metabolic differences between Col-0 and *myb30* plants, PCA was used to find that the first component accounted for 82.3% of total variance (**Figures 5A, B**). Hierarchical clustering of relative metabolite concentrations further demonstrated distinct abundance patterns between Col-0 and *myb30* plants under iron deficiency (**Figure 5C**).

### Metabolic pathway analysis

Furthermore, we conducted a comprehensive analysis of pathways altered in *myb30* plants under iron deficiency using MPA. Twenty-two biological pathways were significantly altered, with the top 10 including aminoacyl-tRNA biosynthesis; purine metabolism; phenylpropanoid biosynthesis; pyrimidine metabolism; arginine and proline metabolism; glutathione metabolism; tryptophan metabolism; diterpenoid biosynthesis; glycerophospholipid metabolism; alanine, aspartate, and glutamate metabolism; and linolenic acid metabolism (**Table 1**).

**Table 1:** Metabolomics pathway analysis in the comparison of WT *vs*. *myb30* seedlings under −Fe conditions.

Pathway names	Pathway IDs	Total	Hits	Raw *P*	−log(*P*)	FDR	Impact
Alanine, aspartate, and glutamate metabolism	ath00250	22	4	0.089669	2.4116	1	0.02874
Pyrimidine metabolism	ath00240	38	5	0.17642	1.7349	1	0.07584
Ascorbate and aldarate metabolism	ath00053	15	2	0.33592	1.0909	1	0
Taurine and hypotaurine metabolism	ath00430	5	1	0.33846	1.0833	1	1
Purine metabolism	ath00230	61	6	0.35188	1.0445	1	0.03068
Butanoate metabolism	ath00650	18	2	0.42378	0.85854	1	0
Indole alkaloid biosynthesis	ath00901	7	1	0.43951	0.8221	1	0
Glycerophospholipid metabolism	ath00564	25	2	0.60207	0.50738	1	0.17957
Diterpenoid biosynthesis	ath00904	26	2	0.62393	0.47172	1	0.18879
Nicotinate and nicotinamide metabolism	ath00760	12	1	0.63009	0.4619	1	0
beta-Alanine metabolism	ath00410	12	1	0.63009	0.4619	1	0.54167
Glycerolipid metabolism	ath00561	13	1	0.65966	0.41604	1	0.21053
Pantothenate and CoA biosynthesis	ath00770	14	1	0.68688	0.37559	1	0.2
Pentose phosphate pathway	ath00030	18	1	0.77584	0.25381	1	0
Tyrosine metabolism	ath00350	18	1	0.77584	0.25381	1	0
Zeatin biosynthesis	ath00908	19	1	0.79384	0.23087	1	0
alpha-Linolenic acid metabolism	ath00592	23	1	0.85261	0.15945	1	0.23
Glutathione metabolism	ath00480	26	1	0.88549	0.12161	1	0
Tryptophan metabolism	ath00380	27	1	0.89475	0.11121	1	0
Arginine and proline metabolism	ath00330	38	1	0.95854	0.042342	1	0
Phenylpropanoid biosynthesis	ath00940	45	1	0.97719	0.023077	1	0.0366
Aminoacyl-tRNA biosynthesis	ath00970	67	1	0.99659	0.003416	1	0

### MYB30 reduces ROS accumulation under iron deficiency stress

**Figure 6:**
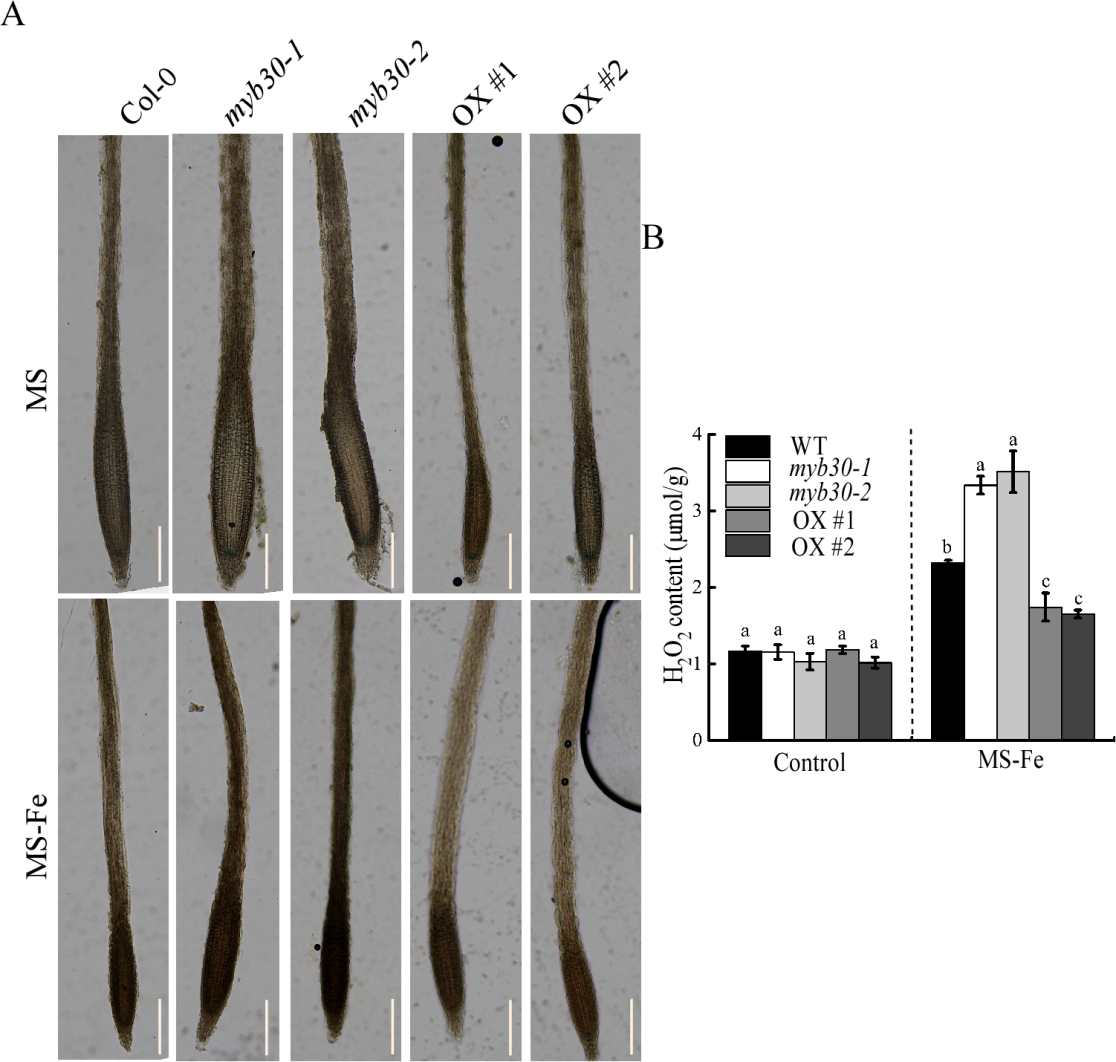
MYB30 is implicated in ROS accumulation under iron-deficient treatment. **(A)** DAB staining when seedlings that were 4 days old were transferred to either control or iron-deficient media for 5 days for further growth. The strength of color showed the concentration of H_2_O_2_ and O_2_^−^. Bar = 200 μm. **(B)** Contents of H_2_O_2_ in seedlings that were relocated to either control or iron-deficient treatment. Lowercase letters above the bars indicated significant differences (*P* < 0.05) by one-way ANOVA. Error bar represents SD (*n* = 3). The experiment was repeated biologically three times.

Metabolic analysis suggested that MYB30 may be involved in antioxidant regulation under iron deficiency. Iron deficiency stress led to ROS generation, which plants counteract via antioxidant defense systems (Gill and Tuteja, 2010; Song et al., 2022). Therefore, we examined the accumulation of ROS in seedlings under iron deficiency by histochemical staining with DAB. Compared to Col-0, *myb30* plants showed more intense DAB staining, whereas MYB30-OX plants exhibited weaker staining, indicating higher and lower ROS accumulation, respectively, under iron-deficient conditions (**Figure 6A**, lower panel). Under normal growth conditions, there were no differences in H_2_O_2_ levels in the tested seedlings by quantitative measurements. However, under iron deficiency, *myb30* plants showed higher accumulation of H_2_O_2_, whereas MYB30-OX plants exhibited lower accumulation of H_2_O_2_, when compared to the level in WT plants (**Figure 6B**). These outcomes revealed that *myb30* plants experienced greater oxidative stress under iron deficiency.

### MYB30 enhances antioxidant enzyme activity under iron deficiency stress

**Figure 7:**
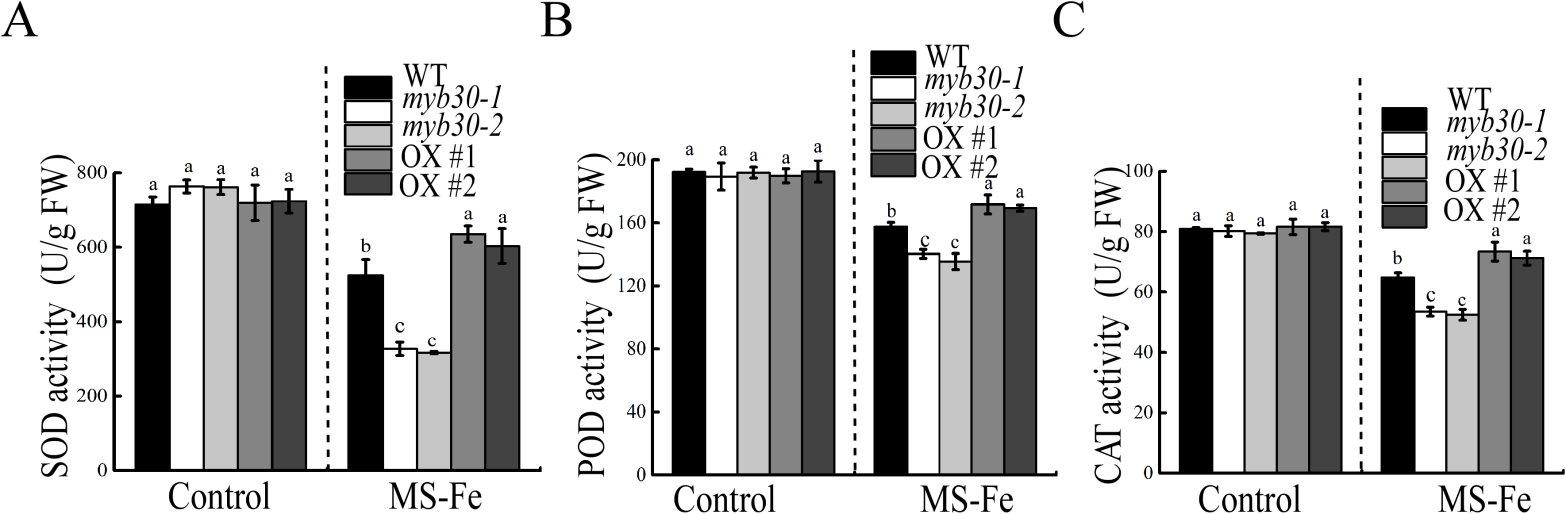
Contents of ROS in seedlings under iron-deficient treatment. The activities of SOD **(A)**, POD **(B)**, and CAT **(C)** in seedlings with or without iron deficiency stress. FW, fresh weight. Lowercase letters above the bars indicated significant differences (*P* < 0.05) by one-way ANOVA. Error bar represents SD (*n* = 3). The experiment was repeated biologically three times.

**Figure 8:**
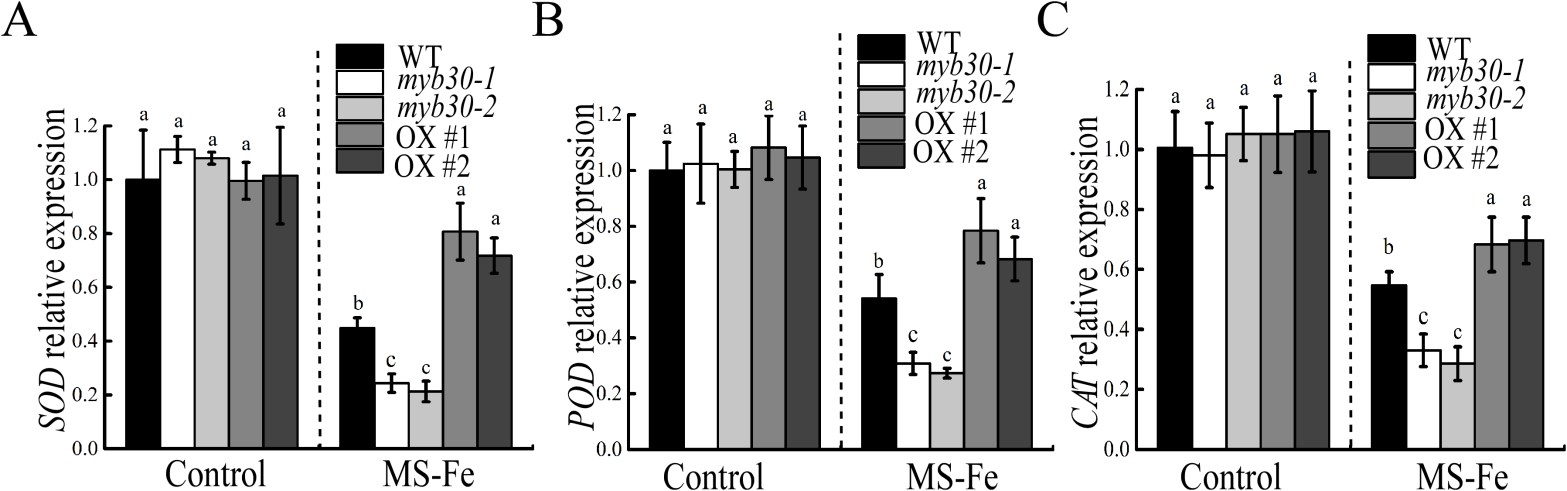
Expression analysis of ROS-responsive genes. RT-qPCR analysis of *SOD***(A)**, *POD***(B)**, and *CAT***(C)** transcript levels in 4-day-old seedlings that were relocated to either control or iron-deficient media for 5 days. The data were adjusted based on gene expression levels in Col-0 under normal conditions. Lowercase letters above the bars indicated significant differences (*P* < 0.05) by one-way ANOVA. Error bar represents SD (*n* = 3). The experiment was repeated biologically three times.

Enzymatic antioxidants are crucial for ROS scavenging. Therefore, the activities of CAT, SOD, and POD in seedlings were assessed. When grown on iron-deficient conditions, all genotypes exhibited reduced antioxidant enzyme activity, with *myb30* mutants showing the lowest activity and MYB30-OX lines showing the highest activity (**Figure 7**). Correspondingly, higher antioxidant enzyme activity was associated with lower H_2_O_2_ accumulation in transgenic plants. RT-qPCR analysis revealed that the expression of *SOD*, *POD*, and *CAT* was significantly lower in *myb30* plants and higher in MYB30-OX plants compared to WT under iron deficiency (**Figure 8**). These results suggested that MYB30 reduces ROS accumulation by upregulating ROS-associated genes and enhancing antioxidant enzyme activity during iron deficiency stress.

### MYB30 enhances the expression of iron deficiency-responsive genes under iron deficiency stress

**Figure 9:**
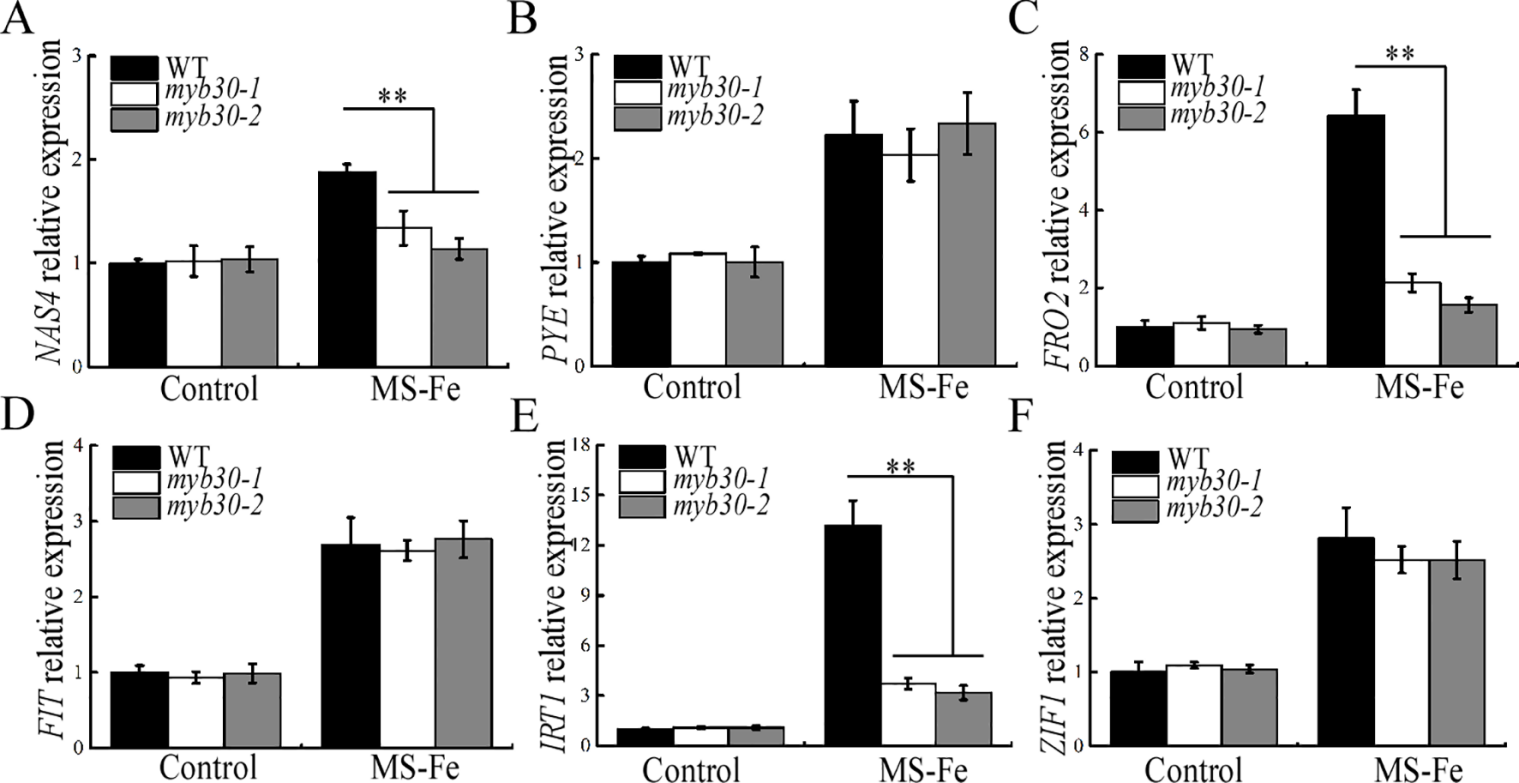
MYB30 affects Fe deficiency-responsive genes. **(A–F)** qRT-PCR analysis of transcript levels in seedlings with or without Fe deficiency stress. *ACTIN2* was used as the internal control. Data were normalized by gene expression level in WT under control conditions. Error bar represents SD (*n* = 3; **P < 0.01, indicating significantly differences); approximately 0.5 g of seedlings per group from one experiment were pooled. Three independent experiments were carried out, and similar results were obtained.

Iron uptake capacity is impaired under iron deficiency, affecting iron homeostasis (Forieri et al., 2017). We measured iron concentrations in seedlings subjected to iron deficiency and found that iron accumulation was significantly reduced in all plants, with *myb30* mutants showing lower iron concentrations than WT (**Supplementary Figure 2**). We also analyzed the expression of key iron uptake and translocation genes, including *NAS4*, *PYE*, *FRO2*, *FIT*, *IRT1*, and *ZIF1* (Colangelo and Guerinot, 2004; Palmer et al., 2013; Brumbarova et al., 2015). Under iron deficiency, the expression of *NAS4*, *FRO2*, and *IRT1* was significantly lower in *myb30* mutants compared to WT (**Figure 9**). These results suggest that *MYB30* orchestrates the expression of genes involved in iron uptake and translocation, thereby enhancing iron accumulation, which is positively associated with enhanced tolerance to iron deficiency.

## Discussion

Iron deficiency triggers transcriptional responses that enhance iron uptake. Among transcription factors regulating iron homeostasis, FIT plays a central role by forming heterodimers with bHLH proteins to directly modulate *FRO2* and *IRT1* expression levels (Sivitz et al., 2011). Previous studies have shown that the *MYB4* gene from *Malus halliana* improves iron deficiency tolerance through promoting photosynthetic pigment synthesis, increasing iron ion content, and enhancing antioxidant enzyme activity (SOD, POD, CAT) (Zhang et al., 2022). Additionally, MYB8 has been implicated in iron deficiency by modulating *IRT1* expression levels in *Arabidopsis* (Gong et al., 2024). Here, we demonstrate that MYB30, a member of the MYB transcription factor family, enhances the plant response to iron deficiency. Phenotypic and biochemical analysis revealed that *myb30* mutants exhibit reduced root length, decreased chlorophyll content, and increased MDA and H_2_O_2_ levels and lower the expression level of *NAS4*, *FRO2*, and *IRT1* gene under iron-deficient conditions, whereas MYB30-OX lines show the opposite trend. Therefore, MYB30 appears to regulate plant responses to iron deprivation. Whether MYB30 interacts with other MYB regulatory factors to affect the expression of iron uptake and transport genes remains to be further investigated.

Metabolomic comparison between the WT and *myb30* seedlings under iron deprivation revealed decreased levels of several metabolites involved in plant defense and antioxidant activity, such as cysteine, tyrosyl-methionine, and 2-hydroxy cinnamic acid. Cysteine is essential for root hair development and plant detoxification (Romero et al., 2014), tyrosyl-methionine exhibits antifungal and antimicrobial activities (Aoki et al., 1995), and cinnamic acid possesses antibacterial and detoxifying properties (Mishra et al., 2020). These reductions suggest compromised defense and antioxidant capacities in *myb30* plants. Conversely, the levels of some metabolites detrimental to plant growth and development, such as 4-nitrophenol, doxylamine succinate, and cepharanthine, were increased. Nitrophenols, a common type of nitroaromatics, are classified as a plant metabolic inhibitor (Fu et al., 2016); doxylamine succinate affects photosynthetic pathways and nitrogen metabolism by disrupting the synthesis of nucleolus ribosomes (Chen and Xiong, 2024); and cepharanthine is a plant-derived alkaloid (Bailly, 2019). Overall, loss of MYB30 function appears to impair antioxidant activity and detoxification capacity under iron deficiency.

MPA facilitates the identification and visualization of altered metabolic pathways using metabolomic data (Schilling et al., 1999). Our analysis revealed changes in stress-responsive pathways such as phenylpropanoid biosynthesis, proline metabolism, tryptophan metabolism, glycerophospholipid metabolism, diterpenoid biosynthesis, and α-linolenic acid metabolism. Tryptophan metabolism is integral to plant immunity (Xia et al., 2021), glycerophospholipids are key regulators of membrane function (Heilmann, 2016), diterpenoids often serve in species-specific chemical defense (Murphy and Zerbe, 2020), and α-linolenic acid metabolism may promote methyl jasmonate synthesis and cold tolerance (Wang et al., 2022). Additionally, disruptions in energy-related pathways such as pyrimidine metabolism and arginine biosynthesis may impair carbohydrate formation and nucleic acid synthesis (Moffatt and Ashihara, 2002), potentially affecting the growth of *myb30* plants under iron deficiency.

ROS homeostasis is essential for cell function, but excess ROS causes oxidative stress and damage (Mhamdi and Van Breusegem, 2018). Iron deficiency increases ROS production, impairing electron transport chains and inducing oxidative stress (Graziano and Lamattina, 2005). In this research, *myb30* mutants accumulated more H_2_O_2_ under iron deficiency, while MYB30-OX plants accumulated less, correlating with MDA levels. To mitigate oxidative damage, plants activate ROS-removing enzymes, including POD, SOD, and CAT (Mittler, 2006). The high activity of those enzymes is associated with low ROS content. We discovered that the activities and gene expression levels of these enzymes were lower in *myb30* plants and higher in MYB30-OX plants compared to WT plants when iron is deficient. These findings imply that MYB30 might influence the response to iron deficiency by altering internal ROS levels. Given the role of ROS in stress signaling (Hammond-Kosack and Parker, 2003), we propose that MYB30 is important for ROS-mediated signal transduction.

Overall, our metabolomic analysis provides insights into the metabolic changes in plants under iron deficiency stress. Loss of MYB30 function weakens antioxidant activity, detoxification capacity, and plant development under iron-deficient conditions. MYB30 alleviates oxidative damage by enhancing antioxidant activity. Future work will focus on identifying the molecular targets of MYB30 in regulating iron deficiency stress and elucidating its regulatory mechanism.

## Data Availability

The original contributions presented in the study are included in the article/**Supplementary Material**. Further inquiries can be directed to the corresponding author.
