# Supplementary figures and images for: Metabolic analysis of MYB30 that regulates iron deficiency stress in Arabidopsis
Source: Front Plant Sci. 2026 Feb 23;17:1756499. doi: 10.3389/fpls.2026.1756499 (PMC12967980; doi:10.3389/fpls.2026.1756499)

72h 48h 24h 0h

Anti-FLAG  
→  
45 KDa

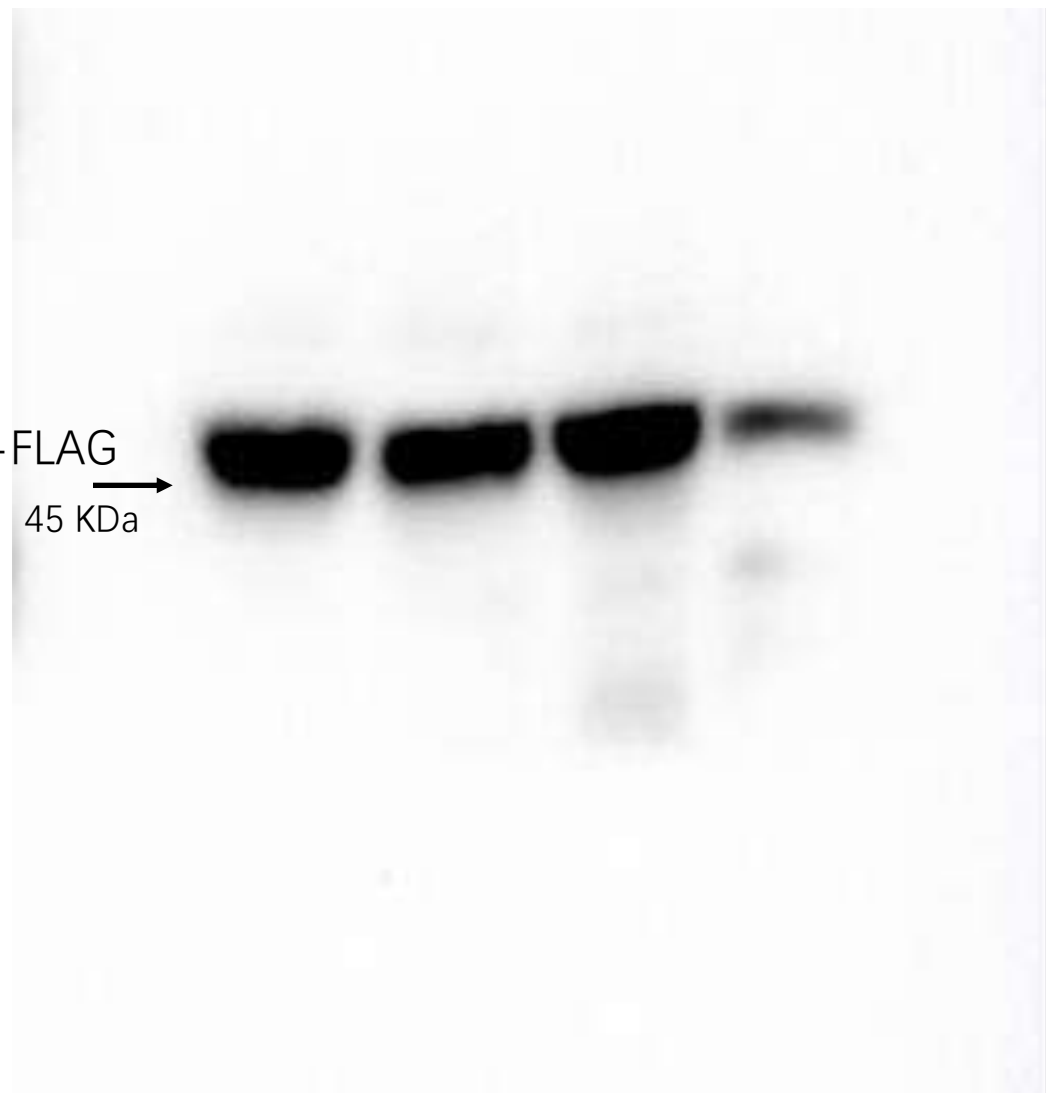

72h 48h 24h 0h

Rubisco

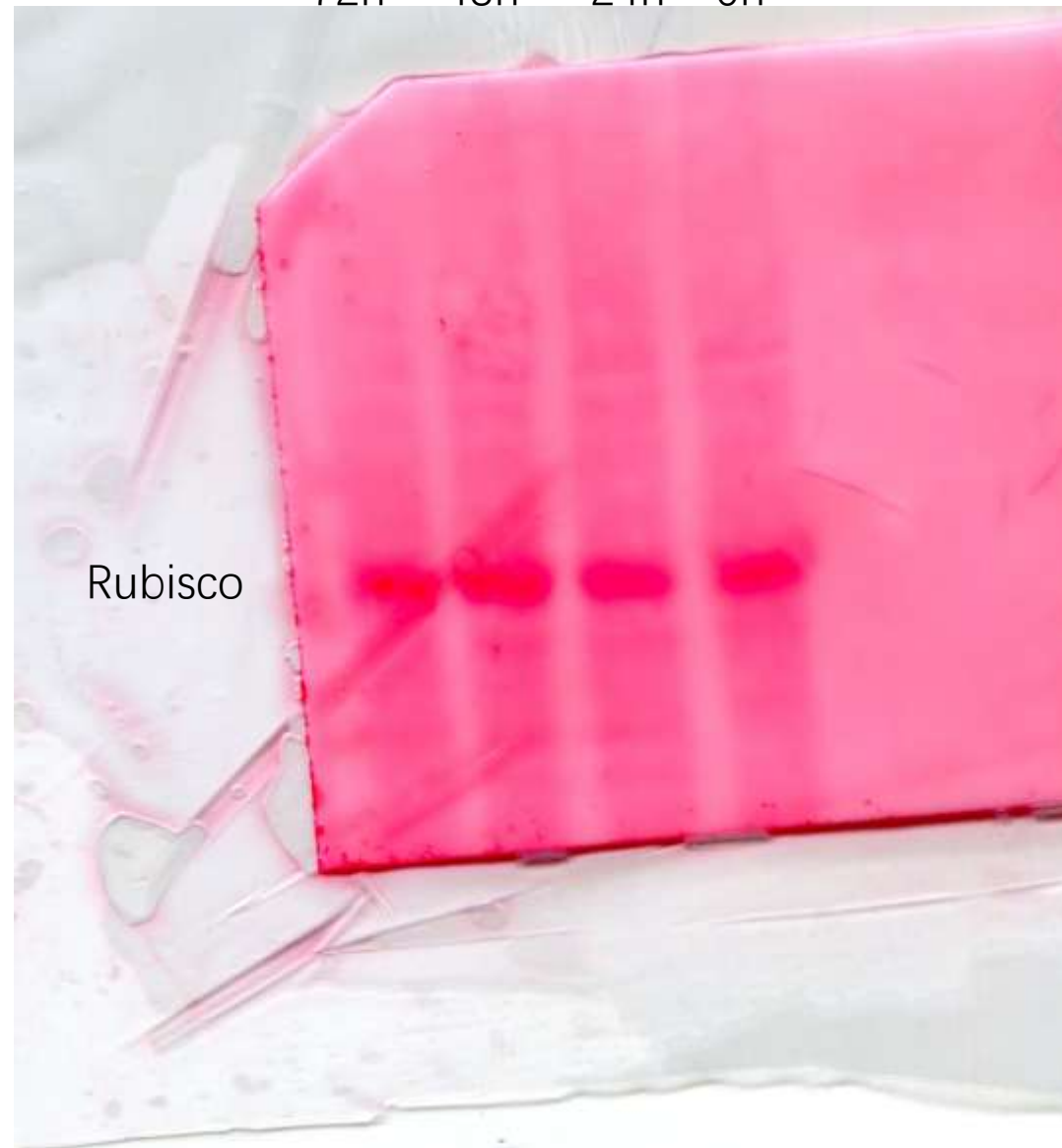

Supplement: Supplementary file 1 [file DataSheet1.pdf]
