# Supplementary material for: Metabolic analysis of MYB30 that regulates iron deficiency stress in Arabidopsis
Source: Front Plant Sci. 2026 Feb 23;17:1756499. doi: 10.3389/fpls.2026.1756499 (PMC12967980; doi:10.3389/fpls.2026.1756499)
Supplement: Supplementary file 2 [file DataSheet2.docx]

**
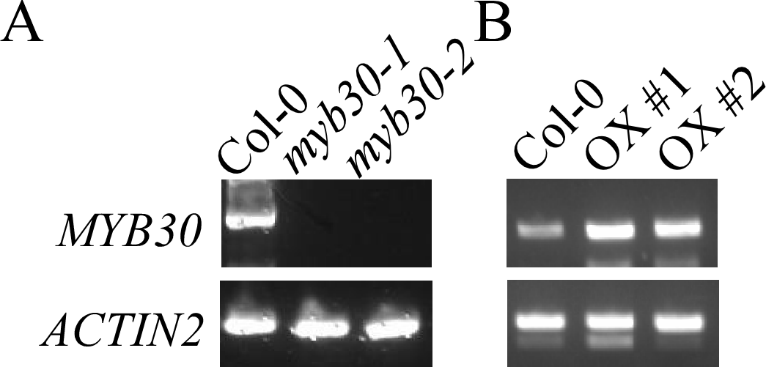
**

**Figure S1. MYB30 transcription level determination.**

(A). Determination of the transcription level of *MYB30* in seedlings of Col-0, *myb30-1* and *myb30-2* using RT-PCR assays.

(B). Determination of the transcription level of *MYB30* in seedlings of Col-0, OX #1 and OX #2 using RT-PCR assays. *ACTIN2* was used as the internal control.


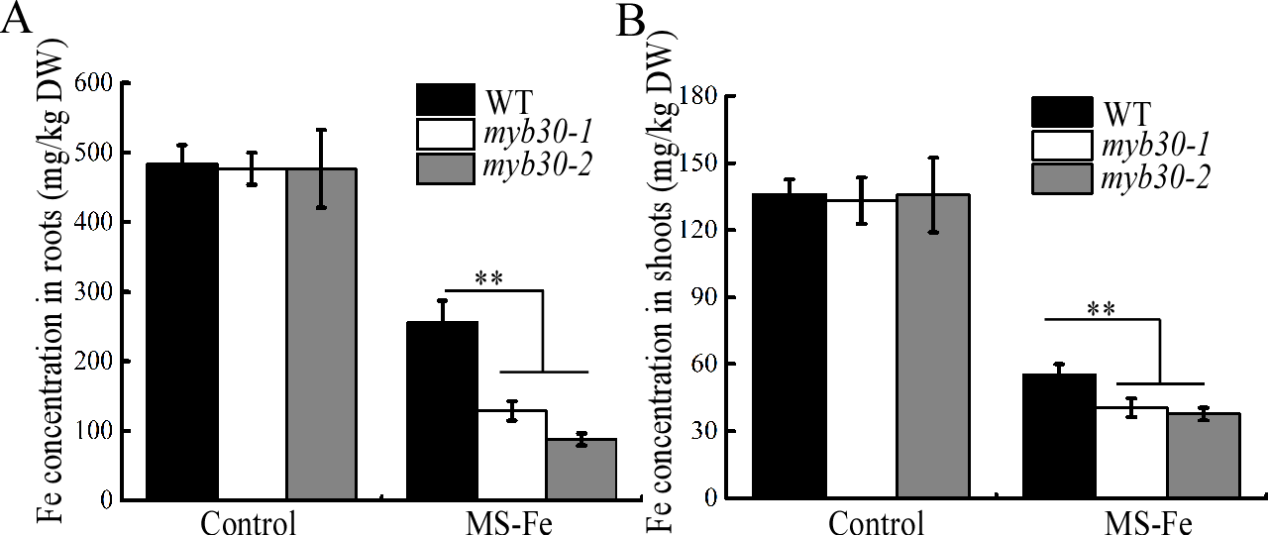


**Figure S2. MYB30 is implicated in Fe homeostasis in response to Fe deficiency.**

(A). Fe concentration determination in plants roots.

(B). Fe concentration determination in plants shoots. Error bar represents SD (n = 3; **P < 0.01, indicating signiﬁcantly differences), about 0.5 g seedlings per group from one experiment were pooled. The experiment was repeated biologically three times. DW, Dry weight.

**Table S1. Primers used in this study.**

| Primer name | Sequence from 5’ site to 3’ site |
| --- | --- |
| LP-MYB30 | AACTGTGCTTCTACGAGCGG |
| RP-MYB30 | CAAGATCCGACCCCGACAAA |
| LP-ACTIN2 | TTGGATCTGTGAACCTCCACT |
| RP-ACTIN2 | CTCTCCATCAAGGTCAAGCCA |
| LP-MYB30-Flag | GCGGTACCATGGGAAGATCACCATGTTGT |
| RP-MYB30-Flag | GGGTCGACGTAACAAGATCCGACCCC |
| LP-CAT | GATCAAATGCCTGTCGGATG |
| RP-CAT | TGAACGAAGAGATTCCACTGC |
| LP-POD | GCCGTTGAAGTTACTGGTGG |
| RP-POD | GTAGCATCAGGAAGACGACC |
| LP-SOD | CAGATTCCTCTTACTGGACCAA |
| RP-SOD | AGCCCTGGAGACCAATGAT |
| LP-RT-NAS4  RP-RT-NAS4  LP-RT-PYE  RP-RT-PYE  LP-RT-FRO2  RP-RT-FRO2 | TGTTCTTGGCTGCTCTTGTAGG  CAAGGCTCAACGATTGGATAGA  CAGGACTTCCCATTTTCCAAG  CTTGTGTCTGGGGATCAGGTT  TCGCCACTATCACTCCTAAATC  CGGTAAGAACAAGAACGCC |
| LP-RT-FIT | CAGTCACAAGCGAAGAAACTCA |
| RP-RT-FIT | AGCAGGAGGATTGATACCGC |
| LP-RT-IRT1 | CTCTTTGCTTCCATCAAATGTTC |
| RP-RT-IRT1 | CCTAACGCTATTCCGAATGG |
| LP-RT-ZIF1  RP-RT-ZIF1 | GCTGTAAGGTGGAGCAGATGAA  TAGTAGAGGAAGGGATAGAGTGAGG |
